# Preparation of CuONPs@PVDF/Non-Woven Polyester Composite Membrane: Structural Influence of Nanoparticle Addition

**DOI:** 10.3390/polym10080862

**Published:** 2018-08-03

**Authors:** Claudio A. Terraza, Rudy Martin-Trasanco, Cesar Saldías, Marjorie González, Ángel Leiva, Alain Tundidor-Camba

**Affiliations:** 1 Research Laboratory for Organic Polymers (RLOP), Faculty of Chemistry, Pontificia Universidad Católica de Chile, Macul 7820436, Chile; cterraza@uc.cl (C.A.T.); ruquim@gmail.com (R.M.-T.); gmarjorie80@gmail.com (M.G.); 2 Energy Research Center. Pontificia Universidad Católica de Chile, Macul 7820436, Chile; 3 Department of Physical Chemistry, Faculty of Chemistry, Pontificia Universidad Católica de Chile, Macul 7820436, Chile; cesaldiasb@gmail.com

**Keywords:** composite membrane, polyvinylidene fluoride, copper oxide nanoparticles, membrane morphology

## Abstract

Membrane distillation techniques have appeared as promising options for guaranteeing the availability of potable water in times of scarcity of this essential resource. For membrane preparation, polyvinylidene fluoride (PVDF) is preferred due to the easier synthesis procedures, with respect to other fluorine-based polymers. In this work, copper oxide nanoparticles (CuONPs) of different weight percent (wt %) embedded in PVDF membranes supported on non-woven polyester fabric (NWPET) were prepared by the phase inversion method, and characterized by spectroscopy (ATR-FTIR, Raman) and electron microscopy techniques (SEM). The PVDF deposited onto the NWPET was mostly composed of its polar β-phase (*F*(β) = 53%), which was determined from the ATR-FTIR spectrum. The *F*(β) value remained constant throughout the whole range of added CuONP concentrations (2–10 wt %), as was determined from the ATR-FTIR spectrum. The absence of signals corresponding to CuONPs in the ATR-FTIR spectra and the appearance of peaks at 297, 360, and 630 cm^−1^ in the Raman spectra of the membranes suggest that the CuONPs are preferably located in the inner PVDF membrane, but not on its surface. The membrane morphologies were characterized by SEM. From the obtained SEM micrographs, a decrease and increase in the amount of micropores and nanopores, respectively, near the surface and intercalated in the finger-like layer were observed. As a result of the CuONP addition, the nanopores in the sponge-like layer decreased in size. The values of water contact angle (WCA) measurements showed a decreasing trend, from 94° to 80°, upon the addition of CuONPs (2–10 wt %), indicating a diminishment in the hydrophobicity degree of the membranes. Apparently, the increase in the amount of nanopores near the surface decreased the membrane roughness, so it became less hydrophobic.

## 1. Introduction

It is expected that, by 2025, the total water shortage will affect 1.8 billion people around the world, and 66% of the total population could be living under water stress conditions. The need to find adequate technologies to supply water and guarantee the livelihood of human beings is a task to accomplish in the short-medium term. Oceans represent ~97% of the global water reserve, and, therefore, water desalinization techniques have attracted attention for fulfilling the water demand in a potable form [1]. Accordingly, the membrane distillation (MD) technique appears to be one of the most promising technologies to obtain potable water from seawater [2].

Membrane distillation is a separation process by which vapor molecules of water, driven by a thermal gradient, pass through a porous hydrophobic membrane [3]. This technique displays several advantages compared to other existing ones (thermal desalinization and reverse osmosis), such as a very high rejection of nonvolatile solute, lower operating temperature and pressure, and the possibility to use low-grade energy sources (e.g., waste heat), as well as the use of renewable energy sources (e.g., solar and geothermal). Not only is MD an economically feasible solution due to the reasons mentioned above, but it is also emerging as an environmentally friendly alternative for water purification.

Considering the characteristics of the separation process, the membranes for MD should have high permeability, low tendency to fouling, high chemical and thermal stability, and a relatively high hydrophobic degree. These features can be achieved by controlling the thickness, porosity, mean pore size, pore-size distribution, geometry, and composition of the membranes. The hydrophobicity is a crucial parameter and should be high enough to withstand a high liquid entry pressure (LEP). This should allow only the water vapor to enter the pores of the membrane, without moistening [1,3].

The most common hydrophobic membranes used for MD are made up of fluoropolymers. From this type of polymer, polytetrafluoroethylene (PTFE) and polyvinylidene fluoride (PVDF) have been widely applied due to their higher mechanical, chemical, and heat resistance in comparison with other hydrophobic materials [1]. Although PTFE is the most hydrophobic, it exhibits low solubility in commons solvents, and membranes fabricated from this polymer must be obtained by stretching or thermal methods, which leads to a relatively low porosity and restricts their processability. Conversely, PVDF is soluble in various common solvents which allow the incorporation of several additives, for the purpose of achieving new properties, and the preparation of hierarchical composite membranes via nonsolvent-induced phase separation (NIPS) process or via phase inversion techniques [4].

Important issues should be considered for the preparation of MD membranes with industrial applications: such challenges include those related to mass flux, heat loss across the membrane, fouling problems, and mechanical strength, which play key roles in the efficiency of the process. Flat-sheet PVDF membranes supported in non-woven polyester fabric (NWPET) yield hydrophilic/hydrophobic layers, which, in addition to accomplishing mass flux increases and avoiding heat loss, confers significant mechanical strength to the membrane [1,3,5].

The modification of roughness and hydrophobicity of the membrane for increasing the efficiency of separation has also been addressed in the past. A direct way to tune these parameters is by incorporating inorganic nanoparticles into the membrane [4,6]. A widely used, simple method to incorporate such types of nanoparticles is their addition into a polymer solution [4,7,8]. Several inorganic nanoparticles, such TiO_2_, SiO_2_, Mg(OH)_2_, Al_2_O_3_, ZnO, CaCO_3_, and CNT, have been incorporated into PVDF membranes [4,9,10,11,12,13,14,15]. In this context, a type of metal nanoparticle that potentially enables improvement of the performance and properties of MD is copper oxide nanoparticles (CuONPs). To the best our knowledge, the use and properties of CuONPs in MD preparation (compared to other types of metal oxide nanoparticles) has been scarcely reported in the literature to date [16,17,18].

CuONPs embedded into PVDF membranes tend to enlarge the surface pores and thicken the finger-like layer [17,18]. These structural membrane features induced an increase in flux by ~150% at a relatively low working temperature (27.5 °C). Interestingly, the incorporation of CuONPs did not dramatically affect membrane selectivity [17]. Recently, PVDF composite membranes using copper oxide nanoparticles and graphene oxide as nanofillers were prepared [18,19]. The obtained composite membrane showed a higher permeation that resulted in finger-like macro-voids and thinner interconnected pores when compared to the non-filled membrane.

Although in the literature reports the preparation of CuONP PVDF composite membranes and PVDF membranes supported onto NWPET, none of the encountered reports deal with the combination of both features. Accordingly, and based on these previous reports, it is probable that the best performance of the thus obtained membranes occurs in direct contact or vacuum membrane distillation setups [17,20].

In the present work, we prepared and characterized a novel CuONP-embedded PVDF composite membrane as the hydrophobic layer, which was supported onto non-woven polyester fabric as the hydrophilic layer. Special emphasis is placed on the presence of the CuONPs, which influenced the size and morphology of the pores in each sublayer of the membrane. Additionally, a correlation between the values of water contact angles with the size of the pores distributed along the membrane was detected. The motivation to carry out the study of the preparation and characterization of these hydrophobic/hydrophilic composite membranes arises from the pursuit of potential alternatives for technological solutions related to the water supply of the future.

## 2. Materials and Methods 

Polydivinylfluoride (PVDF), average *M*_w_~180,000 by GPC, average *M*_n_ ~71,000 beads or pellets, copper(II) sulfate pentahydrate (CuSO_4_·5H_2_O, 98.0%), and *N*,*N*-dimethylformamide (DMF) were purchased from Sigma-Aldrich (Milwaukee, WI, USA) and were used without further purification. Non-woven polyester fabric (NWPET) was purchased from ImportadoraDilaco S.A. (Santiago, Chile).

### 2.1. Preparation of CuONPs

Copper oxide nanoparticles (CuONPs) were prepared using DMF as the reducing and stabilizing agent according to the previous reports in literature [21,22]. Briefly, CuSO_4_·5H_2_O (0.2 g) was poured into a two-necked glass flask containing DMF (10 mL). The flask was connected to a reflux system and heated to 120 °C for 5 h under constant stirring. The solution color turned from a light green to yellowish as the copper oxide nanoparticles were formed. This solution served as stock for the preparation of the doped CuONPs/PVDF membranes at different wt %. A concentration of 20 mg/mL of CuONPs, in terms of copper salt, was used for determining CuONPs/PVDF wt % and the corresponding calculations.

### 2.2. Preparation of Composite NWPET-PVDF Membranes, Neat and Doped with CuONPs

#### 2.2.1. Selection of PVDF Concentration for Preparing the Films

**Figure 1:**
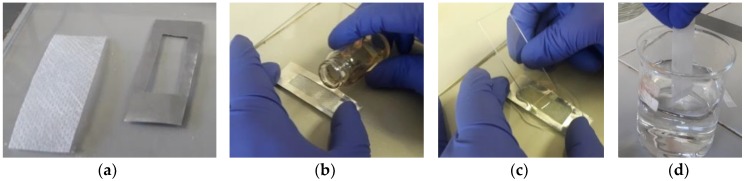
Methodology used for preparing composite membranes. (**a**) Non-woven polyester fabric (NWPET) and alumina template. (**b**) The NWPET was placed on the mold and the polymer solution was added. (**c**) The solution was spread over the NWPET using a slide. (**d**) The NWPET-PVDF was immersed in distilled water for 24 h.

The composite NWPET-PVDF membranes were prepared by the phase inversion method as follows: The casting solutions (1 mL of DMF) at different PVDF concentrations (25, 50, and 200 mg/mL) were stirred for 24 h at room temperature to guarantee a homogeneous polymer solution. The resulting casting solutions were spread onto the NWPET fabric with the help of a hand-made alumina template (~1 mm in thickness) in order to minimize the polydispersity in the film’s thickness (Figure 1). The solutions spread on the NWPET surface were left standing for 20 s before they were immersed for 24 h in distilled water at 25 °C to promote the precipitation of the PVDF. The prepared composite membranes were left to dry in a desiccator with P_2_O_5_ until further use. The dry supported membranes had excellent mechanical stability (without breakage or rolling) due to the high molecular weight of the polymer used. The membrane thickness (NWPET + PVDF) was in the range of 220–230 μm and the NWPET was around 170 μm, therefore, the PVDF active layer was around 60 μm.

#### 2.2.2. Preparation of the NWPET-PVDF Composite Membranes Doped with CuONPs

The CuONPs embedded in the membranes were prepared in a manner similar to that mentioned above, but the PVDF (200 mg) was first dissolved in DMF solutions of CuONPs (1 mL) prepared from the CuONP stock solution (the volumes were adjusted to obtain 2, 4, 6, 8, and 10 wt % CuONPs/PVDF). The previously mixed solutions of PVDF and CuONPs were sonicated for 30 min to guarantee the dispersion of nanoparticles throughout the whole volume. Then, the solutions were spread over the NWPET surface, as was mentioned before.

### 2.3. Membrane Morphology Studies

The membrane morphology was studied using a scanning electron microscope (Zeizz, model EVO MA 10, Oberkochen, Germany). The cross-section SEM micrographs were acquired by fracturing the membranes using liquid nitrogen to freeze them and a surgical scalpel to cut the NWPET. The membranes were coated with gold using a Cressington-108 auto sputter coater (Zeizz, Oberkochen, Germany). The measurements and processes of the obtained SEM micrographs were performed using the free ImageJ (version 1.46 J/Fiji) software from the National Institute of Health, Bethesda, MD, USA [23].

### 2.4. ATR-FT-IR and Raman Spectroscopy

Infrared spectra were recorded on a Perkin-Elmer Spectrum-Two spectrometer (PerkinElmer Inc., Waltham, MA, USA) with a coupled Universal Attenuated Total Reflection (UATR) unit. The deposited polyester PVDF face was directly positioned over the diamond, pressed until reaching 30% of the total supported pressure, and scanned in the range from 4000 to 500 cm^−1^ with a resolution of 1 cm^−1^. The β-phase fraction (*F*(β)) of the different PVDF-covered NWPET was determined from the absorbance of the IR bands at 764 cm^−1^ (*A_α_*) and 840 cm^−1^ (*A_β_*) using Equation (1) [24].

(1) $$\;F\left(\beta \right)=\frac{A_{\beta }}{1.26A_{\alpha }+A_{\beta }}\;$$

Raman Spectra were recorded on a DeltaNu benchtop Raman spectrophotometer with a 785 nm laser. For each sample, 10 spectra were recorded with a 5 s integration time.

### 2.5. Contact Angle Measurements

The contact angle was determined by the sessile drop technique using Dataphysics OCA 20 (DataPhysics, Filderstadt, Germany). A syringe connected to a capillary of Teflon of approximately 2 mm internal diameter was used to deposit the water drop on the samples. All measurements were done at room temperature. The acquisition of the images was carried out by computational processing of the drop profile on the membranes.

## 3. Results and Discussion

### 3.1. Determination of PVDF Concentration to Prepare the Composite Membranes

**Figure 2:**
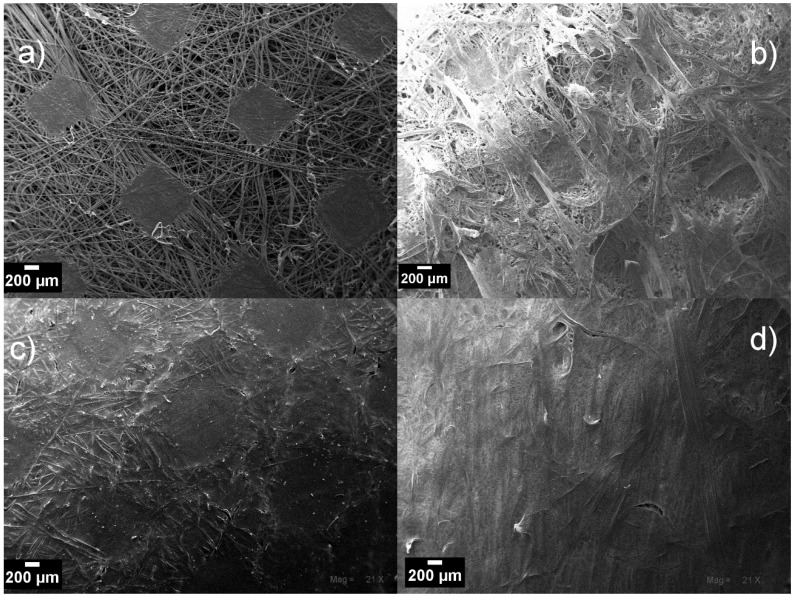
SEM micrographs of: (**a**) neat NWPET fiber; NWPET covered by depositing the casting solution at (**b**) 25 g/L, (**c**) 50 g/L, and (**d**) 200 g/L polyvinylidene fluoride (PVDF) concentrations. (Magnification 21×; scale bar 200 μm.)

The concentration of PVDF solution sufficient to ensure the total coverage of the NWPET surface was determined by spreading polymer solutions of 25, 50, and 200 mg/mL onto the fabric, followed by recording SEM micrographs of the obtained surfaces (Figure 2).

The NWPET fabric is composed of PET fibers randomly aligned and joined by the pressing of the fabric (squares shapes in Figure 2a). As can be noted, by using the lowest PVDF concentration (Figure 2b), a heterogeneous surface morphology was obtained. At this concentration, the polymer amount is not enough to cover all the surface, and certain domains of the PVDF films are seen to be intercalated within the NWPET. When the PVDF concentration is incremented to 50 mg/mL, the surface appears totally covered; however, the pressed fabric pattern and the fiber contours are observed (Figure 2c). This indicates that, despite the film thickness, a homogeneous surface is not observed. At the highest PVDF concentration, the surface is apparently covered (Figure 1d), although some fabric fibers (it is likely that those fibers are far from the pressure zones) still emerge at the surface of films, as detected by ATR-FTIR spectroscopy. Therefore, we considered that this concentration (200 mg/mL) was adequate to prepare the flat-sheet surfaces, as at higher concentrations the viscosity of the medium increased considerably.

### 3.2. ATR-FT-IR and Raman CuO@PVDF Characterization

**Figure 3:**
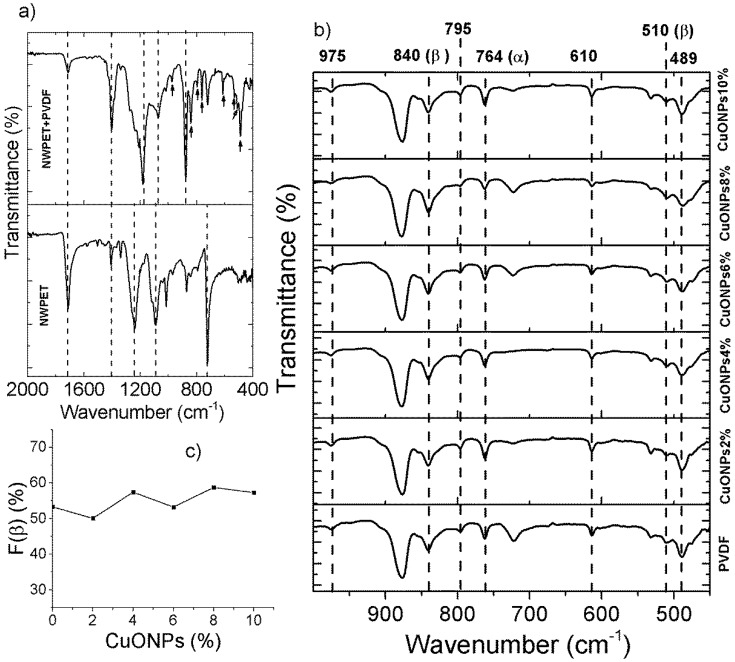
ATR-FTIR spectra of (**a**) neat NWPET and PVDF-covered NWPET and (**b**) NWPET-PVDF membranes prepared at different CuONPs/PVDF compositions. (**c**) Dependence of the β-phase fraction on the CuONPs/PVDF composition.

The PDVF-covered NWPET prepared in absence and presence of CuONPs was characterized by ATR-FT-IR and Raman spectroscopy (Figure 3). Figure 3a shows the ATR-FT-IR spectra of the neat NWPET and the NWPET covered with PVDF films. The FT-IR spectrum of the neat NWPET shows the characteristic peaks of this material at 1713 cm^−1^ (–CO stretching), 1238 cm^−1^ (–C(CO)O– stretching), and 1092 cm^−1^ (–OCC– stretching). When PVDF solution was spread over the NWPET surface, the peaks at 1713 cm^−1^ and 1238 cm^−1^ decrease in intensity, while the peak at 1092 is overlapped by a new intense band at 1182 cm^−1^. This band corresponds to the asymmetric stretch of–CF_2_, while the symmetric component was recorded at 1073 cm^−1^.

The infrared spectrum of PVDF mainly covers the low wavenumber region (Figure 3b). The –CF_2_ wagging (489 cm^−1^) and bending (610 cm^−1^), the skeletal bending (764 cm^−1^), and the –CH_2_ rocking (795 cm^−1^) and twisting (975 cm^−1^) are some of the peaks characteristic of the nonpolar α-phase of PVDF (indicated by black arrows, Figure 3a) [16,25,26]. Two peaks, labeled with black arrows in Figure 3a (at 510 cm^−1^ and 840 cm^−1^, corresponding to the –CF_2_ stretching and –CH_2_ rocking, respectively), were also recorded. These peaks indicate the presence of a β-phase of the PVDF (β-PVDF). The intensity of the peak at 840 cm^−1^ suggests an important contribution of the polar β-PVDF to the polymer structure. Interestingly, in neat PVDF prepared by phase inversion methods, the fraction of the polar β-phase in the polymer structure is very low (*F*(β) < 35%) [13,23]. The *F*(β) value of the polymer deposited onto the non-woven PET indicates that 53% corresponds to the β-phase. These results suggest that the adhesion of the PVDF to NWPET favors the β-phase conformation. Therefore, the observed changes in the intensities of the signals, as well as the presence of others corresponding to the PVDF, help confirm the presence of the polymer on the NWPET surface.

The infrared spectrum of the PVDF films prepared in presence of CuONPs is shown in Figure 3b). Apparently, the presence of CuONPs did not dramatically affect the intensity and wavenumber of the signals (note the signals labeled by dashed lines). To verify the effect of the addition of CuONPs on the crystallinity of the polymer, the fractions of *F*(β) were determined (Figure 3c) for each sample.

It has been reported that the addition of metal oxide nanoparticles to PVDF casting solution causes α- to β-phase conversion [16,26]. As can be noted, the *F*(β) values are similar within the studied CuONP composition range (2–10 wt %). This result could suggest that the crystallinity of the PVDF films is mainly influenced by their deposition onto the non-woven PET and not by the addition of CuONPs. Further indicating the crystallinity’s independence from the CuONPs, no evidence of the nanoparticles was encountered in the ATR-FTIR spectrum. The absence of a peak corresponding to Cu–O strength at 532 cm^−1^ indicates that CuONPs are not present, at least, at the film surface.

**Figure 4:**
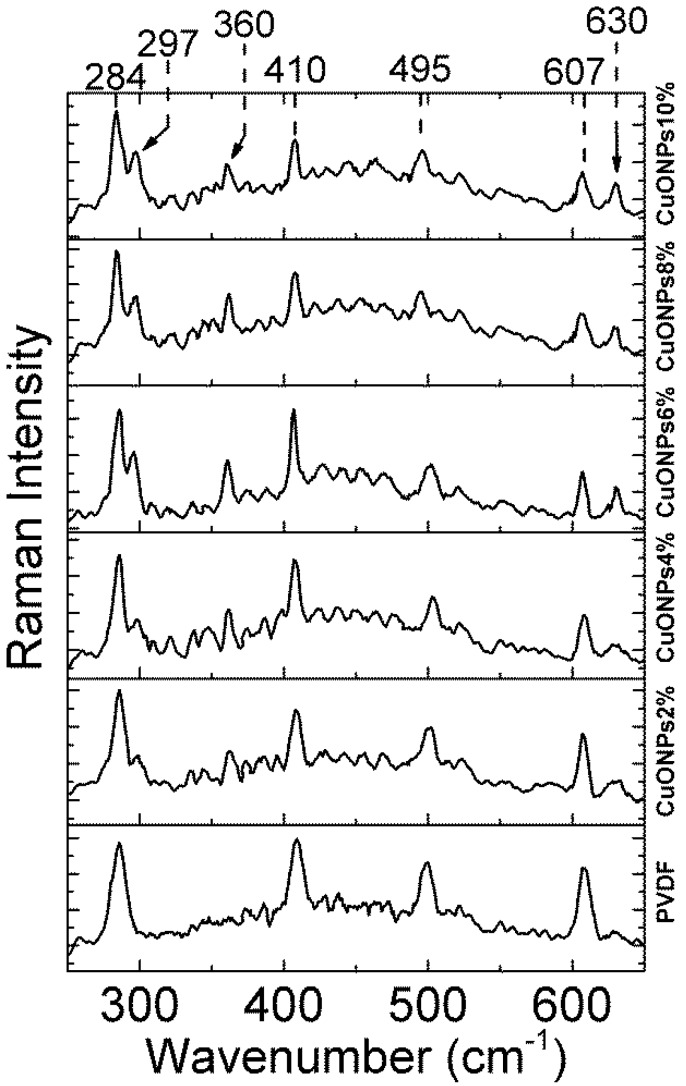
Raman spectra of the NWPET-PVDF membranes prepared at different copper oxide nanoparticles (CuONPs)/PVDF wt %.

In order to explore more deeply into the polymer films and detect the presence of CuONPs, Raman spectra of the PVDF films prepared at different concentrations of CuONPs were recorded. Raman spectra were recorded using a laser of 785 nm wavelength to guarantee its penetration into the sample. The Raman spectrum of the NWPET-PVDF (Figure 4) in the lower wavenumber region shows the characteristic signals of PVDF at 284, 410, 498, and 609 cm^−1^ [25]. The NWPET-PVDF films prepared with CuONPs show three additional peaks at 297, 360, and 630 cm^−1^ (arrows in Figure 4). These peaks are assigned to the three Raman active modes of the CuO (*A_g_ + 2B_g_*), evidencing the presence of the CuONPs embedded into the polymer matrix [27].

Possible explanations of the absence of CuONPs on the NWPET-PVDF surface is their relatively easy dispersibility in water, the precipitation kinetics of the polymer, and the diffusion rate of the CuONPs through the polymer media. The rapid precipitation of the polymer upon immersing the PVDF-impregnated NWPET in water causes the CuONPs near the interface to escape from it. However, the diffusion of CuONPs to the inner PVDF membrane is slow due to the high viscosity of the media, giving rise to the retention of nanoparticles during the precipitation process. It is likely that the CuONPs are present deeper in the pores of NWPET-PVDF membranes [17]. A recent work reported the preparation of graphene oxide/hydroxylated polybenzimidazole composite membranes, in which the crosslinking process of the polymer chains caused the entrapment of the graphene oxide [28]. This approach could be considered for enclosing the CuONPs in the PVDF matrix to prevent the nanoparticles from reaching the bulk solution during the precipitation process.

### 3.3. SEM Characterization

**Figure 5:**
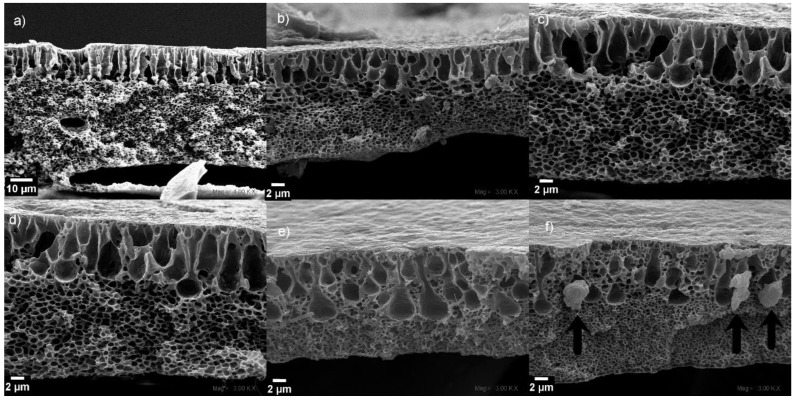
Cross-section SEM micrographs of the PVDF-NWPET samples: (**a**) neat PVDF-NWPET and (**b**–**f**) doped with 2, 4, 6, 8, 10% CuONPs, respectively. The scale bars represent 2 μm, except for (**a**) (0%), which is 10 μm.

To analyze the pore morphologies of the prepared membranes at different CuONP compositions, the corresponding cross-section SEM micrographs were recorded (Figure 5). The membranes are composed of a top PVDF layer attached to a second one formed by the NWPET fabric (Figure 1). The PVDF and NWPET layers exhibit pores with finger-like and sponge-like morphologies, respectively. The size of PVDF and NWPET pores is in the order of micrometers and nanometers, respectively. This pattern was observed in all the prepared membranes (Figure 5).

The finger-like pores of the composite membranes in absence of CuONPs appears aligned and extended to the center of the membrane in a compact distribution. The sponge-like and finger-like porous layers are well defined by a linear boundary along the entire membrane, as can be observed in Figure 5a.

The addition of the CuONPs to the casting solution causes the loss of the above-mentioned linear boundary even at lower concentrations. However, at 2 wt % of the CuONPs, the linear finger-like structures dominate the morphology (Figure 5b). By increasing the CuONP content to 4 wt % and 6 wt % (Figure 5c,d, respectively), the pores adopt a tear-like morphology. Interestingly, at higher amounts of CuONPs (over 6%), the sponge-like layer grows upward to the surface, surrounding and therefore diminishing the number of tear-like pores (Figure 5e). At the highest concentration of CuONPs (10%), the membrane morphology is dominated by the sponge-like structure with some micropores, apparently from the collapsing of the tear-like pores. Additionally, at this concentration, some crystalline structures located at the boundary between the sponge and tear-like pores are observed (Figure 5f, arrows). These structures should correspond to the CuONPs, because a high concentration causes aggregates to diminish the excess of surface energy, relative with their size. It is possible to infer that under the used conditions, this aggregation occurred at concentration higher than 8 wt %, since in the other cross-sectioned SEM micrographs (wt % < 8%), these structures were not observed. Similar results have been reported in mixed composite PVDF membranes prepared in presence of graphene oxide and Cu_x_O (*x* = 1 or 2) nanoparticles [17,18].

**Figure 6:**
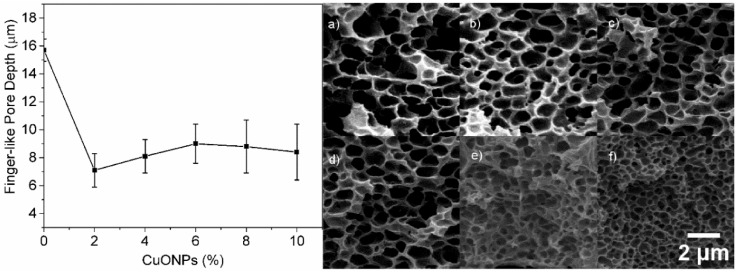
Effect of the CuONP wt % on the depth of the finger-like pores (left panel). SEM micrographs of the sponge-like layer at different CuONP content: (**a**) 0 wt %, (**b**) 2 wt %, (**c**) 4 wt %, (**d**) 6 wt %, (**e**) 8 wt %, and (**f**) 10 wt %. All images have the same size.

The presence of CuONPs not only affected the morphology of the finger-like layer but also their depth, which markedly decreases at the lowest CuONPs content (2%) (Figure 6). The addition of 2% CuONPs causes an increase in the viscosity of the solution [17]. Consequently, the ability of water to penetrate the casting solution and form finger-like pores decreases. This effect is clearly noted with 2% CuONPs. The increase in the CuONP content does not dramatically affect the pore depth (Figure 6, left panel). Additionally, increasing the CuONP amount causes a decrease in both the pore size of the sponge-like structure and the layer thickness (Figure 6, right).

The distribution, morphology, and size of the pores in the PVDF membranes are influenced by the contribution of the thermodynamic and the kinetic factors during the precipitation of the polymer (demixing process). Thermodynamically, the higher the instability of the casting solution, the higher the demixing rate during the phase separation process. Therefore, more finger-like structures and fewer sponge-like structures are formed. From the kinetic perspective, the higher the viscosity of the casting solution, the lower the solvent/nonsolvent exchange rate, which causes retardation of the demixing process. This retardation results in the formation of fewer finger-like structures and more sponge-like structures [29]. Therefore, we suggest that the addition of CuONPs does not affect the stability of the polymer solution (thermodynamic) during the demixing process, but instead affects the precipitation rate (kinetic) due to the increased solution viscosity. Therefore, the membrane structures here obtained are dominated by kinetics and not by thermodynamics of the phase separation process.

### 3.4. Contact Angle Measurements

**Figure 7:**
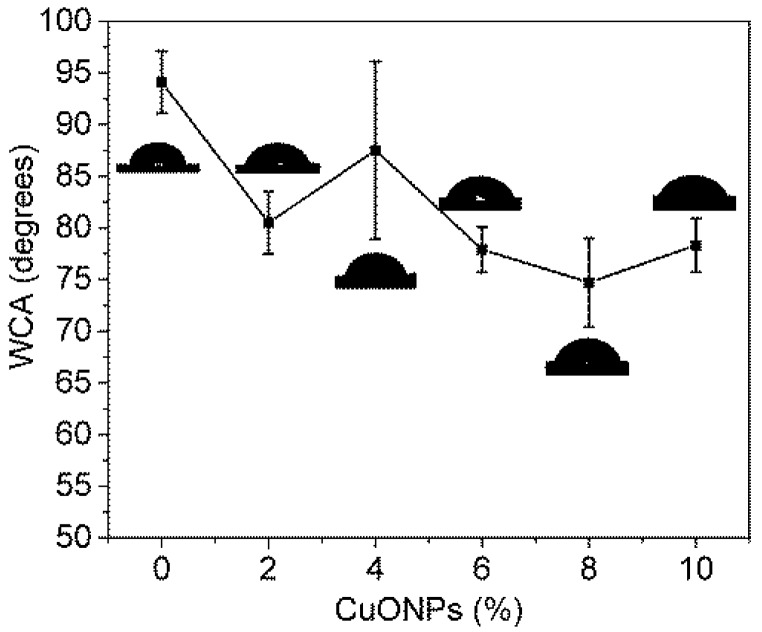
Dependence of the water contact angle with the amount of CuONPs (wt %).

A crucial factor in the efficiency of the MD processes is the membrane hydrophobicity. In a membrane, this parameter depends on its roughness and surface energy [29]. Therefore, the hydrophobicity of the obtained NWPET-PVDF composite membranes was evaluated by water contact angle (WCA) measurements (Figure 7).

In absence of CuONPs, the NWPET-PVDF membrane shows a WCA higher than 90°, a characteristic value for hydrophobic materials. By preparing the membrane with 2 wt % of CuONPs, the WCA decreases by ca. 15 degrees, indicating a less hydrophobic character. Further increase in the CuONP content, i.e., from 4 to 10 wt %, leads to a slow decrease in the WCA.

As was mentioned before, by ATR-FTIR, CuONPs were not detected at the membrane surface. Additionally, the *F*(β) values did not vary with the CuONP wt %. From these two results, we suggest that the decrease in the hydrophobicity is not related to a decrease in the surface energy, but to changes in the roughness of the membranes upon the addition of nanoparticles. 

**Figure 8:**
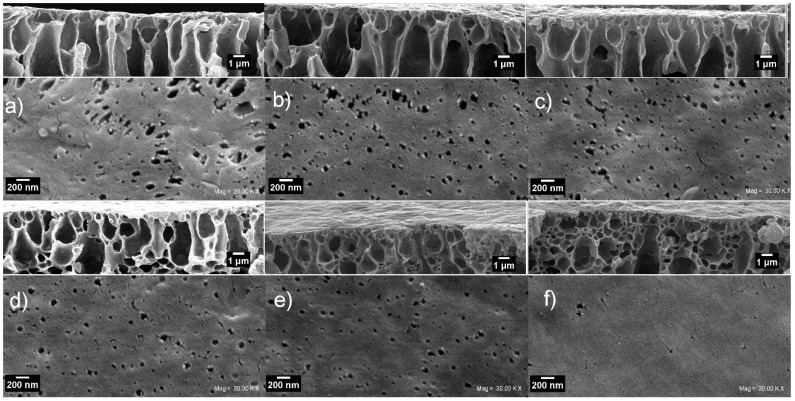
Top and cross-section SEM micrographs of the NWPET-PVDF membranes prepared (**a**) in absence (0 wt %) and (**b**–**f**) in presence of CuONPs (2, 4, 6, 8, and 10 wt %, respectively). Scale bars of top view = 200 nm, cross-section = 1 µm.

Figure 8 shows the surface and top skin cross-section SEM micrograph taken of the NWPET-PVDF membranes prepared in absence (0%) and presence of the CuONPs (2–10 wt %). The neat NWPET-PVDF membrane (Figure 8a) shows a porous and rough surface with pores sizes larger than 200 nm. The addition of 2 wt % CuONPs to the casting solution causes both the surface roughness and the pore sizes of the membrane to decrease (<200 nm) (Figure 8b). The trend to decrease of both parameters continues by increasing the CuONPs wt % from 4% to 10% (Figure 8c–f). At 10 wt % of the CuONPs, a smooth and quasi-absence of pores on the surface was obtained. 

By a simple inspection of the rectangles inset on top of each figure, it is possible to note that the increases of the CuONP wt % lead to a decrease of the micropores extended to the surface and an increase in the amount of nanopores just below the membrane surface. The change in the pore distribution is likely responsible for the decrease of the surface roughness and hence the observed decrease in the WCA measurements upon the addition of CuONPs.

## 4. Conclusions

PVDF membranes, with different wt % of CuONPs embedded, supported on NWPET were prepared by the phase inversion method. The deposition of PVDF solution on NWPET leads to an enhancement of the polar β-phase of the deposited polymer, which does not vary with the amount of CuONPs. Raman spectra suggest that the CuONPs were located in the inner membrane and not on its surface. The addition of the CuONPs to the casting solution causes the loss of the well-defined linear boundary between sponge-like porous and finger-like porous layers. When the CuONP content is between 4 and 6 wt %, the pores adopt a tear-like morphology, but at the highest amount of CuONPs (10%), the membrane morphology is dominated by the sponge-like pores. The resulting membranes become less hydrophobic upon the addition of the nanoparticles. We suggest that the loss in its hydrophobicity arises from the decrease of the membrane roughness and not due to changes in its surface energy. To use these composites in membrane distillation, the hydrophobicity must be increased, which could be obtained by modifying the nanoparticles with hydrophobic molecules.

## References

[1] Drioli E., Ali A., Macedonio F. (2015). Membrane distillation: Recent developments and perspectives. Desalination.

[2] Alkhudhiri A., Darwish N., Hilal N. (2012). Membrane distillation: A comprehensive review. Desalination.

[3] Hou D., Dai G., Wang J., Fan H., Zhang L., Luan Z. (2012). Preparation and characterization of PVDF/nonwoven fabric flat-sheet composite membranes for desalination through direct contact membrane distillation. Sep. Purif. Technol..

[4] Kang G., Cao Y. (2014). Application and modification of poly(vinylidene fluoride) (PVDF) membranes—A review. J. Membr. Sci..

[5] Khayet M., Mengual J.I., Matsuura T. (2005). Porous hydrophobic/hydrophilic composite membranes: Application in desalination using direct contact membrane distillation. J. Membr. Sci..

[6] Warsinger D.M., Swaminathan J., Guillen-Burrieza E., Arafat H.A., Lienhard V J.H. (2015). Scaling and fouling in membrane distillation for desalination applications: A review. Desalination.

[7] Tijing L.D., Woo Y.C., Choi J.-S., Lee S., Kim S.-H., Shon H.K. (2015). Fouling and its control in membrane distillation—A review. J. Membrane Sci..

[8] Qin A., Li X., Zhao X., Liu D., He C. (2015). Engineering a highly hydrophilic PVDF membrane via binding TiO_2_ nanoparticles and a PVA layer onto a membrane surface. ACS Appl. Mater. Interfaces.

[9] Wan H., Briot N.J., Saad A., Ormsbee L., Bhattacharyya D. (2017). Pore functionalized PVDF membranes with in-situ synthesized metal nanoparticles: Material characterization, and toxic organic degradation. J. Membr. Sci..

[10] Gao J., Huang X., Xue H., Tang L., Li R.K.Y. (2017). Facile preparation of hybrid microspheres for super-hydrophobic coating and oil-water separation. Chem. Eng. J..

[11] Dong C., He G., Li H., Zhao R., Han Y., Deng Y. (2012). Antifouling enhancement of poly(vinylidene fluoride) microfiltration membrane by adding Mg(OH)_2_ nanoparticles. J. Membr. Sci..

[12] Ng L.Y., Mohammad A.W., Leo C.P., Hilal N. (2013). Polymeric membranes incorporated with metal/metal oxide nanoparticles: A comprehensive review. Desalination.

[13] Zhang H.Y., Li B.B., Sun D., Miao X.L., Gu Y.L. (2018). SiO_2_-PDMS-PVDF hollow fiber membrane with high flux for vacuum membrane distillation. Desalination.

[14] Smruti R., Sagar R., Somenath M. (2018). Carbon nanotube immobilized membrane with controlled nanotube incorporation via phase inversion polymerization for membrane distillation based desalination. Sep. Purif. Technol..

[15] Li Z., Rana D., Wang Z., Matsuura T., Lan C.Q. (2018). Synergic effects of hydrophilic and hydrophobic nanoparticles on performance of nanocomposite distillation membranes: An experimental and numerical study. Sep. Purif. Technol..

[16] Dutta B., Kar E., Bose N., Mukherjee S. (2015). Significant enhancement of the electroactive β-phase of PVDF by incorporating hydrothermally synthesized copper oxide nanoparticles. RSC Adv..

[17] Baghbanzadeh M., Rana D., Matsuura T., Lan C.Q. (2015). Effects of hydrophilic CuO nanoparticles on properties and performance of PVDF VMD membranes. Desalination.

[18] Zhao C., Lv J., Xu X., Zhang G., Yang Y., Yang F. (2017). Highly antifouling and antibacterial performance of poly (vinylidene fluoride) ultrafiltration membranes blending with copper oxide and graphene oxide nanofillers for effective wastewater treatment. J. Colloid Interface Sci..

[19] Leaper S., Abdel-Karima A., Faki B., Luque-Alled J.M., Alberto M., Vijayaraghavan A., Holmes S.M., Szekely G., Badawy M.I., Shokri N., Gorgojo P. (2018). Flux-enhanced PVDF mixed matrix membranes incorporating APTS-functionalized graphene oxide for membrane distillation. J. Membr. Sci..

[20] Qtaishat M., Rana D., Khayet M., Matsuura T. (2009). Preparation and characterization of novel hydrophobic/hydrophilic polyetherimide composite membranes for desalination by direct contact membrane distillation. J. Membr. Sci..

[21] Pastoriza-Santos I., Liz-Marzán L.M. (1999). Formation and Stabilization of Silver Nanoparticles through Reduction by *N,N*-Dimethylformamide. Langmuir.

[22] Pastoriza-Santos I., Liz-Marzán L.M. (2002). Formation of PVP-Protected Metal Nanoparticles in DMF. Langmuir.

[23] Schneider C.A., Rasband W.S., Eliceiri K.W. (2012). NIH Image to ImageJ: 25 years of image analysis. Nat. Methods.

[24] Sencadas V., Gregorio R., Lanceros-Méndez S. (2009). α to β phase transformation and microestructural changes of PVDF films induced by uniaxial stretch. J. Macromol. Sci. Part B Phys..

[25] Simoes R.D., Job A.E., Chinaglia D.L., Zucolotto V., Camargo-Filho J.C., Alves N., Giacometti J.A., Oliveira O.N., Constantino C.J.L. (2005). Structural characterization of blends containing both PVDF and natural rubber latex. J. Raman Spectrosc..

[26] Kar E., Bose N., Das S., Mukherjee N., Mukherjee S. (2015). Enhancement of electroactive β phase crystallization and dielectric constant of PVDF by incorporating GeO_2_ and SiO_2_ nanoparticles. Phys. Chem. Chem. Phys..

[27] Xu J.F., Ji W., Shen Z.X., Li W.S., Tang S.H., Ye X.R., Jia D.Z., Xin X.Q. (1999). Raman spectra of CuO nanocrystals. J. Raman Spectrosc..

[28] Fei F., Cseri L., Szekely G., Blanford C.F. (2018). Robust Covalently Cross-linked Polybenzimidazole/Graphene Oxide Membranes for High-Flux Organic Solvent Nano filtration. ACS Appl. Mater. Interfaces.

[29] Chen Z., Rana D., Matsuura T., Yang Y., Lan C.Q. (2014). Study on the structure and vacuum membrane distillation performance of PVDF composite membranes: I. Influence of blending. Sep. Purif. Technol..

